# Anti-NMDA Receptor Encephalitis in the Setting of an Immature Ovarian Teratoma: A Case Report and Literature Review

**DOI:** 10.1155/crog/9923415

**Published:** 2025-06-05

**Authors:** Samantha Metzger, Keely Ulmer, Erik Arneson, Christine Gill, Michael J. Goodheart

**Affiliations:** ^1^Department of Obstetrics and Gynecology, University of Iowa Hospitals and Clinics, Iowa City, Iowa, USA; ^2^Department of Neurology, University of Iowa Hospitals and Clinics, Iowa City, Iowa, USA

## Abstract

We report a case of a 19-year-old diagnosed with anti-NMDAR encephalitis in relation to an immature ovarian teratoma. Ultimately, chemotherapy resulted in her improvement despite poor performance status. We provide a literature review of the published cases of anti-NMDAR encephalitis caused by immature teratomas and summarize their treatment.

## 1. Introduction

Anti-N-methyl-D-aspartate receptor (NMDAR) encephalitis is the most common cause of nonviral encephalitis [1]. It presents with new psychosis, seizures, and autonomic instability. NMDAR is commonly associated with the presence of ovarian teratomas, and rarely, the teratomas are malignant (immature teratomas.) [2] Treatment of immature teratomas can include chemotherapy. We report a case of a 19-year-old diagnosed with anti-NMDAR encephalitis in relation to an immature ovarian teratoma. She was initially seen at an outside hospital and was found to have an ovarian mass during a workup for mental status and behavioral changes. Benign gynecology performed a right salpingo-oophorectomy, and the patient was started on high-dose steroids. She was transferred to a tertiary care center for continued treatment due to persistent encephalopathy. Further treatment consisted of IVIG, rituximab, and eventually chemotherapy. Despite a poor performance status, the addition of chemotherapy is what ultimately led to patient improvement. We provide a literature review of the published cases of anti-NMDA encephalitis caused by immature teratomas and summarize their treatment. The primary database used was Pubmed. Search phrases used were “anti-NMDAR encephalitis” and “ovarian teratoma,” and results were filtered for case reports. There were 122 cases of NMDAR related to ovarian teratomas reported, and only 8 of which were cases of immature teratomas.

## 2. Case Report

A 19-year-old woman with a medical history of depression, anxiety, and obsessive-compulsive disorder presented to an outside emergency department with 2 days of confusion, refusal to eat, and extreme behaviors ranging from catatonia to severe agitation and combativeness. Initial differential diagnosis included infection versus primary psychotic disorder versus reaction to doxycycline and prednisone, as she had recently finished a course for an upper respiratory illness. Her workup was largely negative and included a urinalysis, chest radiograph, and respiratory viral panel. A brain MRI was normal. She was started on broad-spectrum antibiotics and acyclovir and admitted for further workup. Lumbar puncture revealed lymphocytic pleocytosis (WBC 33, 92% lymphocytes), but otherwise was negative for extensive serologies and cultures, and so, antibiotics were stopped. Her clinical picture was suspicious for autoimmune encephalitis, and a transvaginal ultrasound revealed a 10-cm complex solid and cystic adnexal mass. Her anti-NMDAR serology was positive in both serum and CSF, and she was started on 1 g solumedrol daily. She was taken to the operating room by benign gynecology for a right salpingo-oophorectomy. Two days later, she was sent to a large tertiary care center for a higher level of care and airway monitoring as she was requiring high doses of lorazepam for her continued extreme behaviors.

Upon arrival, she was unresponsive to pain, hyperreflexic, febrile, and tachycardic. She had an extensive immunological and infectious workup, all of which were negative aside from anti-NMDAR serology. She had features typical for autoimmune encephalitis, such as orofacial automatisms like nasal flaring and characteristic jaw movements and extreme delta brush pattern on EEG. She was started on 400 mg/kg IVIG daily for 5 days in addition to 1 g methylprednisolone daily for 5 days without improvement. Several days later, her pathology returned as a Grade 3 immature teratoma. It was an incompletely staged malignancy with uncertainty regarding surgical spillage, as there was a small amount of hematoma versus tumor contents released during manipulation of the ovary. The gynecologic oncology service was consulted and recommended immediate treatment with etoposide and cisplatin (EP) chemotherapy for four cycles, holding bleomycin as pulmonary function testing could not be completed with the current cognitive state. Per immunology recommendations, the patient was also given 1 g of rituximab with an additional dose to follow in 2 weeks.

The patient continued to have extreme behaviors, ranging from catatonia to severe agitation. Despite poor performance status, she was given her first cycle of EP 2 days after she was seen by the gynecologic oncology service. She was also given rituximab that day. Within 2 days, her signs of autonomic instability were improving, and she was asking appropriate questions about her hospital stay. She became neutropenic, which resolved. She completed a second cycle of EP inpatient. She continued to show marked improvement, and tube feedings were stopped. She was discharged home with plans for outpatient occupational and physical therapy. She presented outpatient for her third and fourth cycles of bleomycin, etoposide, and cisplatin (BEP). Bleomycin was added for the third and fourth cycles, as she had normal pulmonary function testing. The patient has made a complete recovery and has returned to life as normal for her. She is being treated with rituximab every 6 months for maintenance therapy.

## 3. Discussion

Anti-NMDAR encephalitis is commonly associated with ovarian teratomas. NMDA receptors are present in the hippocampus, and it is believed that ectopic neural tissues present in the teratoma can cause the immune system to produce antibodies against the NMDA receptor. This results in encephalitis [2]. Symptoms typically begin with a nonspecific prodromal phase consisting of headaches, nausea, fevers, etc. Within days to weeks, psychiatric and behavioral symptoms develop. Neurologic symptoms such as seizures, involuntary movements, memory deficits, autonomic instability, and decreased consciousness quickly follow [11, 12]. Treatment primarily consists of tumor removal and immune therapy. First-line immune therapy includes steroids, IVIG, and plasmapheresis. Second-line immune therapy is used in refractory cases and includes rituximab and cyclophosphamide. Second-line therapies such as rituximab can also be used for long-term maintenance in refractory cases [13].

Rarely, anti-NMDAR encephalitis is caused by a malignant immature teratoma. To our knowledge, there are only eight reported cases of immature teratomas causing anti-NMDAR encephalitis (Tables 1 and 2). In addition to the typical treatment regimens as outlined above, chemotherapy has contributed to positive outcomes in the treatment of NMDAR encephalitis in the setting of immature teratomas. Chemotherapy was used in the treatment of 5 of the above cases (Table 2), in addition to our current case. BEP is the standard of care chemotherapy regimen for immature teratoma [14]. BEP was used in two of the reported cases [7, 10]. EP were given in two other cases, but bleomycin was held due to the inability to perform the necessary pulmonary function [8], as was the case for our patient initially, and for tracheobronchitis [6]. Another reported patient did not have surgery but was treated with paclitaxel, ifosfamide, and cisplatin [9]. All of the other patients had been treated with immune therapy and surgery but made a complete recovery after chemotherapy. To the best of our knowledge, there is no reported recurrence of anti-NMDAR encephalitis in the setting of immature ovarian teratoma after treatment.

Prompt diagnoses and treatment of anti-NMDAR encephalitis lead to better outcomes [15]. Anti-NMDAR encephalitis is commonly linked to ovarian teratomas, but very rarely are those teratomas malignant. We have presented a case of anti-NMDAR encephalitis in the setting of an immature teratoma and summarized the treatment regimens of other published cases. Through this, we have illustrated the benefit of prompt treatment with chemotherapy in the treatment of NMDAR encephalitis in the setting of immature teratomas that have not responded to the typical first- and second-line treatments. Treatment teams must consider chemotherapy in patients with immature teratomas who have not responded to initial treatments, even despite a poor performance status, as that is what ultimately contributed to this patient's improvement.

## Figures and Tables

**Table 1:** Immune therapy and surgery for immature teratomas.

**Year**	**Author**	**Age**	**Treatment**	**Outcome**
2011	Reyes-Botero et al. [3]	Unknown	Surgery, steroids, and IVIG	Complete recovery
2017	Mutti et al. [4]	31	Surgery, steroids, IVIG, and plasmapheresis	Complete recovery
2021	Imataka and Yoshihara [5]	13	Surgery, IVIG, and steroids	Complete recovery

**Table 2:** Chemotherapy in addition to immune therapy (with or without surgery) for immature teratomas.

**Year**	**Author**	**Age**	**Treatment**	**Outcome**
2012	Dizon et al. [6]	22	Surgery, steroids, plasmapheresis, etoposide, and cisplatin	Complete recovery
2012	Tanyi et al. [7]	34	Surgery, steroids, IVIG, plasmapheresis, 3 cycles of bleomycin, etoposide, and cisplatin	Complete recovery
2020	Nizam et al. [8]	19	Surgery, steroids, IVIG, plasmapheresis, and 4 cycles of etoposide and cisplatin (no bleomycin because unable to complete PFT)	Complete recovery
2021	Chaudhuri et al. [9]^a^	29	Steroids, plasmapheresis, rituximab, and chemo (paclitaxel, ifosfamide, and cisplatin)	Complete recovery
2021	Chetram et al. [10]	Early 20s	Surgery, methylprednisolone, and 4 cycles of bleomycin, etoposide, and cisplatin	Complete recovery
2024	Metzger et al.	19	Surgery, methylprednisolone, IVIG, rituximab, and 2 cycles of etoposide and cisplatin plus 2 cycles of etoposide, cisplatin, and bleomycin	Complete recovery

^a^This was the only case that did not include surgery in the treatment of the immature teratoma.

## Data Availability

The data that support the findings of this study are available from the corresponding author upon reasonable request.
